# Supplementary material for: The interactome of intact mitochondria by cross-linking mass spectrometry provides evidence for coexisting respiratory supercomplexes
Source: Mol Cell Proteomics. 2017 Dec 8;17(2):216–32. doi: 10.1074/mcp.RA117.000470 (PMC5795388; doi:10.1074/mcp.RA117.000470)
Supplement: Supplemental Data [file supp_RA117.000470_133922_0_supp_23978_fzffwf.zip › spectra_annotation/mito_spectra_annotation/449-1-8-1-9-1.pdf]

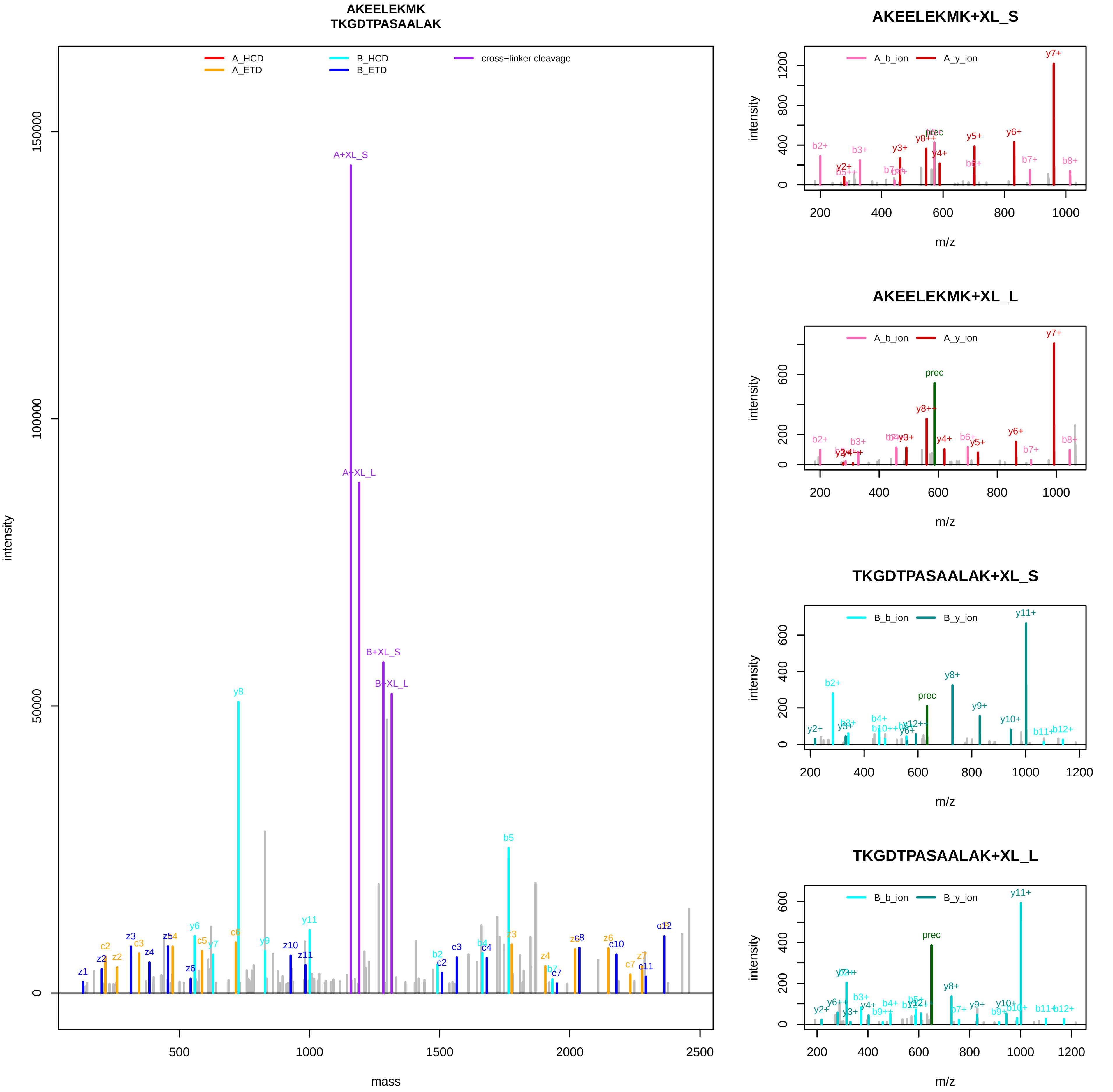

AKEELEKMK
TKGDTPASAALAK
A_HCD
A_ETD
B_HCD
B_ETD
cross−linker cleavage
intensity
mass
0
50000
100000
150000
500
1000
1500
2000
2500
A+XL_S
A+XL_L
B+XL_S
B+XL_L
y8
b5
y11
y6
y7
y9
c6
c5
c4
c3
c2
c7
c8
c10
c11
c12
z1
z2
z3
z4
z5
z6
z7
z10
z11
b2
b4
b7
AKEELEKMK+XL_S
AKEELEKMK+XL_L
TKGDTPASAALAK+XL_S
TKGDTPASAALAK+XL_L
A_b_ion
A_y_ion
B_b_ion
B_y_ion
prec
m/z
intensity
y2+
y3+
y4+
y5+
y6+
y7+
y8++
b2+
b3+
b6+
b7+
b8+
y9+
y10+
y11+
y12++
b11+
b12+
